# Association of Body Mass Index With Mortality and Clinical Outcomes in Patients With Nonalcoholic Fatty Liver Disease: A Medical Information Mart for Intensive Care IV (MIMIC-IV) Cohort Study

**DOI:** 10.7759/cureus.110031

**Published:** 2026-06-01

**Authors:** Miracle C Chukwuma, Abidemi O Akinrinmade, Dhakshayani G Nath, Grace A Gbigbi-Jackson, Redvers Kadzungwa, Nkechi R Ihezie, Adewale A Kuye, Ifunanya R Ekeocha, Adaeze E Uzozie, Anulika Anyata

**Affiliations:** 1 Internal Medicine, Albany Medical College, Albany, USA; 2 Internal Medicine, State University of New York (SUNY) Upstate Medical University, Syracuse, USA; 3 Medicine and Surgery, Benjamin S. Carson School of Medicine, Babcock University, Ilishan-Remo, NGA; 4 Internal Medicine, American University of Barbados, Bridgetown, BRB; 5 Family Medicine, Medical University of South Carolina (MUSC) Health Florence Medical Center, Florence, USA; 6 Epidemiology and Public Health, Boston University School of Public Health, Boston, USA; 7 Medicine, Allied Physicians Group, New York, USA; 8 Medicine, Windsor University School of Medicine, Cayon, KNA; 9 Public Health, Imperial College London, London, GBR; 10 Internal Medicine, Nnamdi Azikiwe University Teaching Hospital, Nnewi, NGA; 11 General Medicine, University of Nigeria Teaching Hospital, Enugu, NGA; 12 Community Medicine, Alex Ekwueme Federal University Ndufu-Alike, Abakaliki, NGA

**Keywords:** body mass index, mimic-iv, mortality, nonalcoholic fatty liver disease, obesity, retrospective cohort

## Abstract

Background: Nonalcoholic fatty liver disease is a common condition linked to metabolic disorders and increasing healthcare burden. The relationship between body mass index (BMI) and clinical outcomes in this population remains uncertain, with prior studies reporting mixed findings.

Objective: This study aimed to evaluate the association between BMI and in-hospital mortality among adults with nonalcoholic fatty liver disease, with BMI analyzed as a binary variable (underweight or normal weight vs overweight or obese).

Methods: This retrospective cohort study used data from the Medical Information Mart for Intensive Care IV (MIMIC-IV) version 3.1 database. Adult patients aged 18 years or older with nonalcoholic fatty liver disease were identified using the International Classification of Diseases (ICD) codes, and only the first hospital admission was included. BMI was calculated from recorded height and weight and categorized as underweight or normal weight versus overweight or obese. Multivariable logistic regression was used to estimate adjusted odds ratios for in-hospital mortality. Due to the low number of mortality events, a parsimonious model was applied to reduce overfitting, including age, sex, race, coronary artery disease, and chronic kidney disease as covariates.

Results: A total of 2,955 patients were included. In-hospital mortality was low, with 15 deaths observed. Overweight or obese BMI was not significantly associated with mortality compared to underweight or normal-weight BMI (adjusted odds ratio: 0.42; 95% CI: 0.14-1.27; p=0.124). Increasing age was associated with higher mortality (adjusted odds ratio: 1.05 per year; 95% CI: 1.01-1.10; p=0.024). No significant associations were observed for sex, race, coronary artery disease, or chronic kidney disease.

Conclusion: BMI was not associated with in-hospital mortality in patients with nonalcoholic fatty liver disease. Age was associated with increased mortality risk. These findings support the need to consider broader clinical factors beyond BMI in risk assessment.

## Introduction

Nonalcoholic fatty liver disease (NAFLD) is one of the most common chronic liver diseases in the world, indicating a large burden of metabolic conditions such as obesity, type 2 diabetes, and dyslipidemia [1]. NAFLD is defined by an excess of fat in the liver without excessive alcohol use and includes a range of diseases from simple steatosis (the accumulation of fat in the liver) to nonalcoholic steatohepatitis (NASH), fibrosis, cirrhosis, and liver cancer [2,3]. Because of the increasing incidence of obesity, NAFLD is a growing concern to public health due to the significant impact it has on people who are ill and accessing medical care [4,5].

Body mass index (BMI), a common measure of how much body fat a person has, is a major factor in the development of NAFLD and how NAFLD develops over time [6]. Obesity has been shown to be a risk factor for hepatic steatosis because of its relationship to insulin resistance, chronic inflammation, and altered lipid metabolism [7]. However, the relationship between BMI and clinical outcomes in NAFLD is complex and not clearly understood [8]. Generally, people with a higher BMI tend to have poorer metabolic profiles and more severe disease than those with a lower BMI; however, some studies suggest that overweight/mildly obese individuals may have improved survival than normal-weight/low-weight individuals in some healthcare settings (the so-called "obesity paradox") [9,10]. This raises a number of important questions regarding the prognostic implications of BMI in patients with NAFLD, particularly in critically ill populations [11].

NAFLD can occur in people with a normal BMI and/or low BMI, particularly if they are genetically predisposed to the disease, have visceral fat, and/or have been metabolically dysregulated [12]. These people are often referred to as "lean NAFLD" patients and may have unique disease characteristics and disease trajectories [13]. Therefore, BMI alone will not provide a complete picture of risk heterogeneity in the NAFLD population [14]. Therefore, understanding how BMI groups impact clinical outcomes (mortality, length of stay, complications) is important for improving risk stratification and determining how best to manage them clinically [15].

Patients with NAFLD frequently have multiple comorbidities, including cardiovascular disease (CVD), diabetes mellitus, and chronic kidney disease, all of which contribute to adverse patient outcomes [12]. These comorbidities may influence prognosis through complex interactions with BMI in acute and critically ill inpatients [16]. For example, underweight inpatients may have lower physiological reserve and be more susceptible to acute illness, while severely obese inpatients may have difficulties related to respiratory function, hemodynamics, and pharmacologic management [17].

While there is a growing body of literature supporting the notion that NAFLD is a significant contributor to the overall global disease burden, there is still insufficient evidence on the independent relationship between BMI and short-term clinical outcomes in hospitalized patients with NAFLD [18]. Most of the published literature examining the relationship between BMI and clinical outcomes in NAFLD has focused on long-term liver-related clinical outcomes or has been conducted in settings other than acute care (e.g., outpatient) [19].

The study will employ the Medical Information Mart for Intensive Care IV (MIMIC-IV) database. The MIMIC-IV database allows researchers to fill in these gaps by creating a large, heterogeneous sample of hospitalized patients who have substantial records with complete clinical, lab, and outcomes data recorded [20].

This study aimed to evaluate the association between BMI and in-hospital mortality among adult patients with NAFLD using a retrospective cohort from the MIMIC-IV database, with BMI categorized as a binary variable to account for the low number of mortality events and improve model stability. The study will contribute to a better understanding of the prognosis of BMI with NAFLD and help assist in clinical decision-making for managing this condition that is literally exploding in number.

## Materials and methods

Study design and data source

This study used a retrospective cohort design based on data obtained from the MIMIC-IV version 3.1 database. This is a large, publicly accessible critical care database that contains deidentified clinical information for patients admitted to intensive care units at Beth Israel Deaconess Medical Center in Boston, Massachusetts [21]. Access to the dataset was obtained through Google BigQuery following the completion of the required training, credentialing, and data use agreements. Data extraction was performed using structured query language to retrieve patient-level information from relevant tables within the database, including admissions, patient demographics, diagnostic codes, and available derived clinical variables.

Study population

The study population included adult patients aged 18 years or older with a diagnosis consistent with NAFLD, identified using the International Classification of Diseases (ICD) Ninth and Tenth Revision codes recorded within the diagnoses table of the MIMIC-IV database. Specifically, NAFLD was identified using ICD-9 code 571.8 and ICD-10 code K76.0. These codes are assigned during routine clinical care and stored as part of hospital administrative records.

Only the first hospital admission for each patient was included to avoid duplication and ensure independence of observations. Patients were required to have documented BMI values derived from recorded height and weight measurements available in the database. Records with implausible or inconsistent anthropometric values were excluded during data cleaning. Implausible anthropometric values were defined as BMI values less than 10 kg/m² or greater than 70 kg/m². After applying these criteria and validating extracted variables, the final analytic sample consisted of 2,955 patients.

Variables and measures

The primary exposure variable was BMI, calculated as weight in kilograms divided by height in meters squared (kg/m^2^). BMI was categorized using the standard World Health Organization clinical thresholds as underweight (<18.5 kg/m²), normal weight (18.5-24.9 kg/m²), overweight (25.0-29.9 kg/m²), and obese (≥30.0 kg/m²). Due to the low number of mortality events, BMI categories were collapsed into a binary variable to avoid sparse cell counts and improve the statistical stability of the regression model. The primary outcome was in-hospital mortality, defined using the hospital expiration flag recorded in the admissions table of the MIMIC-IV database. This variable indicates whether a patient died during the index hospitalization and is derived from hospital administrative records within the database. Mortality was coded as a binary variable, with a value of 1 indicating in-hospital death and 0 indicating survival to discharge. The use of this variable is consistent with established MIMIC-IV studies, as it provides a standardized and validated measure of in-hospital mortality. The number of mortality events observed in the cohort was low, with 15 deaths among 2,955 patients, and this was taken into account during model specification and interpretation.

Covariates included age at admission, sex, race, admission type, diabetes, hypertension, chronic kidney disease, and coronary artery disease. Race was categorized as White, Black, and Other, with Asian, Hispanic, and other groups combined into the Other category to reduce sparse cells. Admission type was grouped into emergency, observation, and elective categories based on clinical classification. All comorbidities were defined using diagnosis codes and treated as binary variables.

Missing data

After data extraction and preprocessing, observations with missing data on variables included in the analysis were excluded using a complete-case approach. As a result, the final analytic dataset contained no missing values for the variables used in the regression models.

Statistical analysis

Descriptive statistics were used to summarize baseline characteristics. Continuous variables were assessed for approximate normality using distributional summaries and were presented as means with standard deviations. Given the large sample size, parametric methods were considered appropriate, and categorical variables were presented as counts and column percentages. Group comparisons by the outcome variable were conducted using two-sample t-tests for continuous variables and chi-squared tests for categorical variables. Multivariable logistic regression analysis was performed to estimate adjusted odds ratios and 95% confidence intervals for the association between BMI category and in-hospital mortality. Due to the low number of mortality events, a parsimonious model was constructed to avoid overfitting, and BMI categories were collapsed into a binary variable. Race categories were also simplified to reduce sparse data bias. Multicollinearity among independent variables was assessed using variance inflation factors, with values ranging from 1.01 to 1.15 and a mean variance inflation factor of 1.08, indicating no evidence of significant multicollinearity. Statistical significance was defined as a two-sided p-value of less than 0.05. All data processing and statistical analyses were performed using Stata version 18 (StataCorp LLC, College Station, TX, USA) [22].

Ethical considerations

The MIMIC-IV database contains fully deidentified patient data, and all users must complete the required training and data use agreements prior to access. As the dataset does not contain identifiable patient information, this study was exempt from institutional review board approval. All analyses were conducted in accordance with relevant data use guidelines and ethical standards for research involving deidentified data.

## Results

Table 1 below presents the baseline characteristics of the study population stratified by BMI category.

**Table 1:** Baseline characteristics by BMI category (n=2,955) Continuous variables are presented as mean (SD). Categorical variables are presented as n (percent) using column percentages. Group comparisons were performed using two-sample t-tests for continuous variables and chi-squared tests for categorical variables. The asterisk (*) indicates statistical significance at the 0.05 level. BMI: body mass index The table was generated by co-authors using Stata version 18 (StataCorp LLC, College Station, TX, USA) [22].

Variable	Underweight/normal weight (n=451)	Overweight/obese (n=2,504)	Test statistic	P-value
Age, years, mean (SD)	56.32 (15.08)	52.46 (14.37)	t=5.21	<0.001*
BMI in kg/m^2^, mean (SD)	22.13 (2.25)	34.61 (7.03)	t=-37.36	<0.001*
Length of stay, days, mean (SD)	6.19 (8.77)	4.76 (6.45)	t=4.06	<0.001*
Gender, n (%)				
Female	224 (49.67)	1,338 (53.43)	χ²=2.18	0.140
Male	227 (50.33)	1,166 (46.57)		
Race, n (%)				
White	301 (66.74)	1,618 (64.62)	χ²=2.26	0.323
Black	51 (11.31)	349 (13.94)		
Other	99 (21.95)	537 (21.44)		
Admission type, n (%)				
Emergency	216 (47.89)	878 (35.06)	χ²=46.94	<0.001*
Observation	175 (38.80)	942 (37.62)		
Elective	60 (13.31)	684 (27.32)		
Diabetes, n (%)				
No	311 (68.96)	1,535 (61.30)	χ²=9.55	0.002*
Yes	140 (31.04)	969 (38.70)		
Hypertension, n (%)				
No	268 (59.42)	1,326 (52.96)	χ²=6.44	0.011*
Yes	183 (40.58)	1,178 (47.04)		
Chronic kidney disease, n (%)				
No	401 (88.91)	2,230 (89.06)	χ²=0.01	0.928
Yes	50 (11.09)	274 (10.94)		
Coronary artery disease, n (%)				
No	382 (84.70)	2,158 (86.18)	χ²=0.69	0.405
Yes	69 (15.30)	346 (13.82)		

The findings show that patients in the underweight or normal-weight group were older than those in the overweight or obese group, with a mean age of 56.32 (15.08) years compared to 52.46 (14.37) years (p<0.001). The mean BMI differed substantially between groups (22.13 (2.25) kg/m^2^ versus 34.61 (7.03) kg/m^2^; p<0.001). Length of stay was longer in the underweight or normal-weight group (6.19 (8.77) days) compared to the overweight or obese group (4.76 (6.45) days) (p<0.001). There was no statistically significant difference in gender distribution, with females comprising 224 (49.67%) and 1,338 (53.43%) in the two groups (p=0.140). Race distribution was also similar between groups (p=0.323). Admission type differed significantly (p<0.001), with a higher proportion of emergency admissions in the underweight or normal-weight group, 216 (47.89%), compared to the overweight or obese group, 878 (35.06%), while elective admissions were more frequent in the overweight or obese group, 684 (27.32%), compared to the underweight or normal-weight group, 60 (13.31%). Diabetes was more common in the overweight or obese group (969 (38.70%)) compared to the underweight or normal-weight group (140 (31.04%)) (p=0.002). Hypertension was also more frequent in the overweight or obese group compared to the underweight or normal-weight group (1,178 (47.04%) versus 183 (40.58%); p=0.011). There were no significant differences in chronic kidney disease (p=0.928) or coronary artery disease (p=0.405).

Table 2 below presents the multivariable logistic regression analysis examining the association between BMI category and in-hospital mortality.

**Table 2:** Multivariable logistic regression analysis for in-hospital mortality Adjusted odds ratios are reported with 95% CI. The model was adjusted for age, gender, race, coronary artery disease, and chronic kidney disease. The asterisk (*) indicates statistical significance at the 0.05 level. BMI: body mass index The table was generated by co-authors using Stata version 18 (StataCorp LLC, College Station, TX, USA) [22].

Variable	Adjusted odds ratio (95% CI)	P-value
BMI category		
Overweight/obese vs underweight/normal weight	0.42 (0.14-1.27)	0.124
Age (per year)	1.05 (1.01-1.10)	0.024*
Gender		
Female vs male	0.62 (0.21-1.81)	0.381
Race		
Black vs White	1.77 (0.47-6.71)	0.400
Other vs White	0.33 (0.04-2.57)	0.289
Coronary artery disease (yes vs no)	1.19 (0.35-4.04)	0.775
Chronic kidney disease (yes vs no)	1.56 (0.45-5.41)	0.458

The results indicate that overweight or obese BMI was not significantly associated with in-hospital mortality compared to underweight or normal-weight BMI (adjusted odds ratio: 0.42; 95% CI: 0.14-1.27; p=0.124). Increasing age was associated with higher odds of mortality, with an adjusted odds ratio of 1.05 per year (95% CI: 1.01-1.10; p=0.024). Gender was not significantly associated with mortality, with females having an adjusted odds ratio of 0.62 compared to males (p=0.381). Race was not significantly associated with mortality, with adjusted odds ratios of 1.77 for Black patients and 0.33 for Other races compared to White patients (p=0.400 and p=0.289), respectively. Coronary artery disease and chronic kidney disease were not significantly associated with mortality, with adjusted odds ratios of 1.19 and 1.56, respectively, both with p-values greater than 0.05.

**Figure 1:**
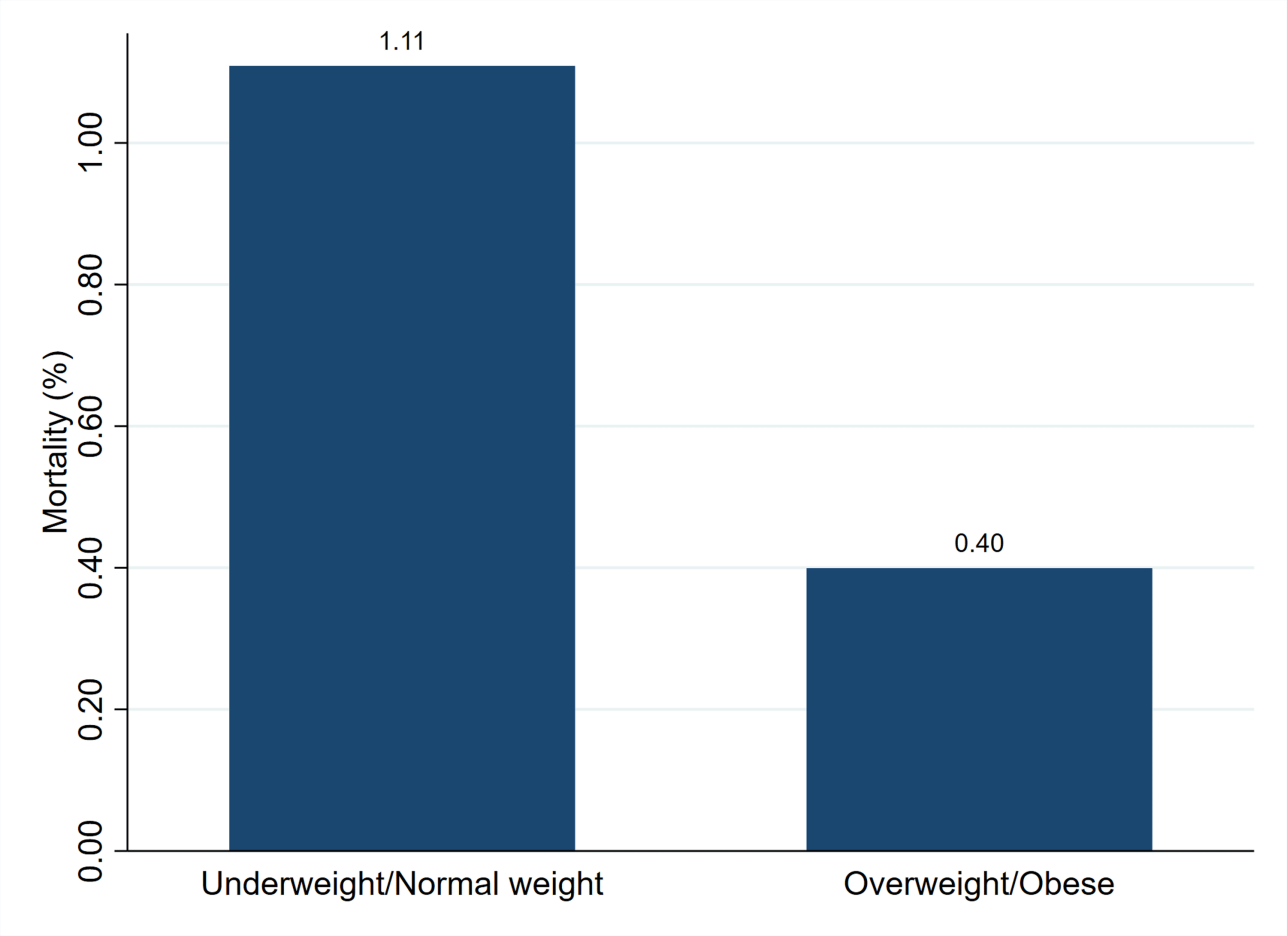
In-hospital mortality by BMI category BMI: body mass index

Figure 1 below illustrates the in-hospital mortality rate by BMI category.

The results indicate that the mortality rate was higher in the underweight or normal-weight BMI group, approximately 1.11%, compared to the overweight or obese group, 0.40%. The difference in proportions suggests a lower observed mortality rate among patients with higher BMI. These findings should be interpreted with caution due to the low number of mortality events, which may affect the stability and precision of the observed differences.

## Discussion

This study examined the relationship between BMI and in-hospital mortality among patients with NAFLD using a large clinical dataset. The findings show that overweight or obese BMI was not significantly associated with in-hospital mortality when compared to underweight or normal-weight BMI. Age was the only variable that showed a statistically significant association with mortality, with increasing age linked to higher odds of death. In addition, patients in the underweight or normal-weight group were older and had longer hospital stays, while diabetes and hypertension were more common in the overweight or obese group. These results are consistent with prior work showing that BMI alone may not fully capture risk in patients with NAFLD, particularly in the setting of metabolic dysfunction [6,7]. Although some prior studies have described a possible "obesity paradox," where overweight or obese individuals may have similar or lower mortality in certain clinical settings, the findings in this study were not statistically significant and should be interpreted cautiously. Given the low number of mortality events and wide confidence intervals, the results are better considered exploratory and do not provide sufficient evidence to support or refute this hypothesis [8,10,11]. At the same time, the presence of NAFLD in individuals with normal BMI has been described as a distinct clinical entity, often associated with metabolic abnormalities and increased risk despite lower body weight [12-14]. This may help explain why the underweight or normal-weight BMI group in this study showed higher age and longer hospital stay. However, this observation should be interpreted with caution, as differences in age distribution, comorbidity burden, and potential frailty among older patients may contribute to the observed outcomes. These factors may confound the relationship between BMI and mortality, suggesting that lower BMI does not necessarily indicate lower clinical risk in this population.

Although obesity remains a major risk factor for NAFLD, current clinical guidance recommends that the evaluation of patients with NAFLD should extend beyond BMI alone and include the assessment of associated metabolic and systemic comorbidities. The American Association for the Study of Liver Diseases (AASLD) recommends evaluation for conditions such as hypertension, diabetes, insulin resistance, hypothyroidism, obstructive sleep apnea, and polycystic ovary syndrome when assessing patients with NAFLD [23,24]. In the present study, patients in the underweight or normal-weight BMI group were older, experienced longer hospital stays, and had a greater proportion of emergency admissions despite having lower rates of diabetes and hypertension. These findings suggest that lower BMI does not necessarily indicate lower clinical risk among hospitalized patients with NAFLD and reinforce the need for comprehensive clinical assessment rather than reliance on anthropometric measures alone. In addition, NAFLD may remain clinically significant even in the absence of obesity, particularly among individuals with underlying metabolic dysfunction or age-related physiological vulnerability [12,14].

Given the limitations of liver biopsy, including invasiveness, sampling variability, procedural risk, and cost, noninvasive approaches for fibrosis assessment are increasingly incorporated into routine clinical practice [24]. Several serum-based fibrosis scores, including the Fibrosis-4 (FIB-4) index, aspartate aminotransferase-to-platelet ratio index (APRI), enhanced liver fibrosis index, and NAFLD fibrosis score, are commonly used for initial risk stratification [25,26]. Imaging-based modalities such as vibration-controlled transient elastography, shear wave elastography, and magnetic resonance elastography also provide noninvasive assessment of liver fibrosis severity [25]. Among these methods, FIB-4 and transient elastography are widely used because of their accessibility, reproducibility, and cost-effectiveness in identifying patients at risk for advanced fibrosis. Although magnetic resonance elastography demonstrates high diagnostic accuracy, its use remains limited in many clinical settings because of cost and availability constraints [25]. These approaches may improve the identification of high-risk NAFLD patients whose clinical risk may not be adequately reflected by BMI alone.

Current clinical guidance in the United States emphasizes the importance of identifying metabolic risk factors rather than relying solely on BMI when evaluating patients with NAFLD. Clinical management focuses on the reduction of weight, control of diabetes, and treatment of cardiovascular risk factors as central components of care [2,3]. These recommendations reflect the understanding that NAFLD is closely linked to metabolic dysfunction, including insulin resistance and systemic inflammation [7]. The findings of this study support this approach, as diabetes and hypertension were more prevalent in the overweight or obese group, yet these patients did not demonstrate higher mortality. This suggests that clinical outcomes may be influenced by a broader range of metabolic and systemic factors rather than BMI alone. The growing burden of NAFLD globally further highlights the need for risk stratification strategies that go beyond simple anthropometric measures [4,5].

Several biological mechanisms may contribute to the observed patterns in this study. Obesity is associated with increased adipokine production, chronic low-grade inflammation, and insulin resistance, all of which are implicated in the progression of NAFLD [7]. NAFLD is strongly linked to metabolic abnormalities such as central obesity, dyslipidemia, hypertension, hyperglycemia, and ongoing liver function disturbances [2]. Understanding the complex interaction of these metabolic abnormalities is important in understanding the pathophysiological mechanisms linking BMI and NAFLD outcomes. Furthermore, the lack of significant association between BMI and NAFLD suggests that other metabolic abnormalities, other than obesity, may be playing a huge role in the development of NAFLD outcomes.

Obesity is closely related to insulin resistance which results in hepatic fat accumulation leading to poor outcomes among patients with NAFLD. Insulin resistance appears to be the central mechanism linking elevated BMI to NAFLD outcomes. In obese individuals, impaired insulin signaling promotes increased lipolysis and elevated circulating free fatty acids, which are subsequently taken up by hepatocytes, leading to triglyceride accumulation and hepatic steatosis. Under this pathway, the development of NAFLD occurs via a two-step process [2]. The first step of this process is fat deposition in the liver that will result in increased insulin resistance. The second part of the process is cellular and molecular changes involving oxidative stress and oxidation of fatty acids in the liver [2].

However, patients with lower BMI may have underlying metabolic dysfunction that is not reflected in body weight alone, including altered lipid metabolism or genetic predisposition [12,14]. Differences in nutritional status, muscle mass, and physiological reserve may also play a role, particularly in hospitalized populations. Prior studies have shown that lower BMI in critically ill patients may be associated with worse outcomes in certain settings, which may relate to reduced energy reserves during acute illness [17]. In contrast, some studies have reported that severe obesity may be associated with worse outcomes in liver disease, indicating that the relationship between BMI and outcomes is complex and may vary depending on disease severity and patient characteristics [15]. These findings highlight that BMI should be interpreted within the broader clinical context rather than as an isolated predictor.

Strengths and limitations of the study

This study has several strengths, including the use of a large, well-characterized clinical database and a clearly defined cohort of patients with NAFLD. The availability of detailed clinical variables allowed for the adjustment of key confounders in the analysis.

There are also important limitations that should be considered. The retrospective design limits the ability to establish temporal relationships, and the study relies on coded clinical data, which may introduce misclassification. Although there was no missing data in the final analytic dataset, certain clinically relevant variables such as laboratory values, liver disease severity measures (e.g., fibrosis stage), medication use, and lifestyle factors were not included, which may influence outcomes.

The extremely low number of mortality events (n=15) substantially limits statistical power, increases the risk of imprecise estimates, and reduces the ability to detect meaningful associations. As a result, the findings should be interpreted as inconclusive rather than definitive evidence of no association between BMI and mortality.

In addition, BMI was analyzed as a binary variable to address sparse data, which may have resulted in loss of clinically relevant heterogeneity across BMI categories. Important confounders, including measures of illness severity, metabolic parameters, and detailed comorbidity burden, were not fully captured, which may contribute to residual confounding.

Sensitivity analyses were not performed due to the low number of mortality events and limited statistical power, which may further limit the assessment of the robustness of the findings.

Race was categorized using a simplified grouping structure, which may not fully capture heterogeneity across populations. The absence of more granular classifications, including Hispanic and other non-Hispanic subgroups, may limit the ability to detect important differences in risk profiles and outcomes across diverse populations.

Additionally, the interpretation of longer hospital stay and higher age observed in the underweight or normal-weight BMI group should be made with caution. These findings may reflect underlying differences in patient characteristics, including comorbidity burden, disease severity, or frailty among older individuals, rather than a direct relationship between BMI category and outcomes.

The study was based on data from a single healthcare system, which may limit generalizability. Future research should include larger and more diverse cohorts with higher event rates, incorporate detailed clinical and laboratory measures, and apply more granular risk stratification approaches to better characterize outcomes in patients with NAFLD.

## Conclusions

This study highlights that BMI was not significantly associated with in-hospital mortality among patients with NAFLD, while increasing age was linked to higher mortality risk. These findings suggest that BMI alone may not adequately reflect clinical risk in this population. The results support the need to focus on broader metabolic and clinical factors when assessing hospitalized patients with NAFLD. From a clinical perspective, reliance on BMI as a primary risk marker may be insufficient. Future research should include larger and more diverse populations, incorporate additional clinical and laboratory measures, and further examine the role of metabolic dysfunction in shaping outcomes among patients with NAFLD.
